# Improved Antioxidant and Mechanical Properties of Food Packaging Films Based on Chitosan/Deep Eutectic Solvent, Containing Açaí-Filled Microcapsules

**DOI:** 10.3390/molecules28031507

**Published:** 2023-02-03

**Authors:** Barbara E. Teixeira-Costa, Willian Hermogenes Ferreira, Francisco M. Goycoolea, Brent S. Murray, Cristina T. Andrade

**Affiliations:** 1 Programa de Pós-Graduação em Biotecnologia-PPGBIOTEC, Faculdade de Ciências Agrárias, Universidade Federal do Amazonas, Avenida General Rodrigo Otávio 6200, Manaus 69077-000, AM, Brazil; 2 Programa de Pós-Graduação em Ciência de Alimentos-PPGCAL, Instituto de Química, Universidade Federal do Rio de Janeiro, Avenida Moniz Aragão 360, Bloco 8G/CT2, Rio de Janeiro 21941-594, RJ, Brazil; 3 School of Food Science and Nutrition, University of Leeds, Leeds LS2 9JT, UK

**Keywords:** polyelectrolyte complexes, *Euterpe oleracea*, bioactive coatings, choline chloride, deep eutectic solvent, food packaging, thin films, DPPH, microcapsules, elongation at the break

## Abstract

The development of biobased antioxidant active packaging has been valued by the food industry for complying with environmental and food waste concerns. In this work, physicochemical properties for chitosan composite films as a potential active food packaging were investigated. Chitosan films were prepared by solution casting, plasticized with a 1:2 choline chloride: glycerol mixture as a deep eutectic solvent (DES) and incorporated with 0–10% of optimized açaí oil polyelectrolyte complexes (PECs). Scanning electron microscopy and confocal laser scanning microscopy revealed that the chitosan composite films were continuous and contained well-dispersed PECs. The increased PECs content had significant influence on the thickness, water vapor permeability, crystallinity (CrD) and mechanical and dynamic behavior of the films, as well as their antioxidant properties. The tensile strength was reduced in the following order: 11.0 MPa (control film) > 0.74 MPa (5% DES) > 0.63 MPa (5% DES and 5% PECs). Films containing 2% of PECs had an increased CrD, ~6%, and the highest elongation at break, ~104%. Films with 1% of PECs displayed the highest antioxidant properties against the ABTS and DPPH radicals, ~6 and ~17 mg TE g^−1^, respectively, and highest equivalent polyphenols content (>0.5 mg GAE g^−1^). Films with 2% of particles were not significantly different. These results suggested that the chitosan films that incorporated 1–2% of microparticles had the best combined mechanical and antioxidant properties as a potential material for food packaging.

## 1. Introduction

Packaging is a fundamental barrier to contain, protect and promote the conservation, and extension of food shelf-life. Natural biopolymers have been used as good renewable materials for the development of edible food packaging [1,2]. The use of natural polymers has advantages because they contribute to the reduction in the use of petroleum-derived plastics and environmental pollution [3]. Natural polymers are known for their bioavailability, biocompatibility, biodegradability, low toxicity and low allergenicity [4,5]. Polysaccharides, proteins and their combinations have been used for the development of packaging membranes [6,7,8,9]. Chitosan is amongst those polymers that have been used for the design of sustainable packaging [9]. Chitosan is a linear polysaccharide derived from chitin deacetylation and is mainly obtained from crustacean shells, although it can also be extracted from fungal and insect sources [10,11,12]. Chemically, chitosan is a random copolymer composed by acetylated and deacetylated *D*-glucosamine units linked by *β*-1,4 glycosidic bonds. The repeating units contain hydroxyl groups and amine groups, and the latter are responsible for the polycationic behavior of chitosan under acidic conditions [4].

Biopolymer films, including those made from chitosan, are generally characterized by pronounced stiffness; compatible plasticizers can be incorporated to overcome this drawback. The plasticizers increase the matrix free volume and molecular mobility of amorphous biopolymers, minimizing intermolecular hydrogen bonding interactions [13]. Deep eutectic solvents (DES) have been recommended as sustainable solvent systems for biopolymer film plasticization because of their physical-chemical properties and biodegradability [14,15]. They are obtained by complex formation between a hydrogen bond donor (HBD) and a quaternary ammonium salt (hydrogen bond acceptor–HBA) [16]. These mixtures present many advantages, such as being chemical and thermally stable, non-flammable, and having low volatility, melting point and toxicity, all of which makes DES a good choice for plasticizing food packaging [12,17,18]. Studies suggest that the bioaccumulation of DES in the environment is very low, log K_ow_ values < 1. Thus, this result indicates a lack of accumulation tendency [15,19]. The mixture of choline chloride and glycerol (ChCl-Gly) has been used for chitosan-based films [17,20], particularly at a molar ratio of 1:2 [21,22]. Some DES were also used as food-grade Maillard-type flavor modulators, such as *N*^2^-(1-carboxyethyl) guanosine 5′-monophosphate, *N*-(1-methyl-4-oxoimidazolidin-2-ylidene) aminopropionic acid and 1-deoxy-d-fructosyl-*N*-*β*-alanyl-l-histidine, indicating their application for improving sensorial properties in foods [23].

Food packaging can be developed for different purposes, such as passive packaging, active packaging (intelligent or smart packaging) and sustainable or green packaging [24]. It can also be incorporated with bioactive additives, which interact with foods to improve their quality and shelf-life [25]. Different natural substances, such as polyphenols, carotenoids and polyunsaturated fatty acids, can be used for preserving purposes, and may also display beneficial properties for consumers’ health [26]. However, the efficiency of bioactive substances in promoting health benefits decreases considerably when they are exposed to oxidative conditions, such as air, humidity, heat and light. Encapsulation in biopolymeric matrices is a good way to maintain their biological activities [27,28]. Complex coacervation is the most widely used technique for encapsulating hydrophobic substances, such as vegetable oils. This technique produces particles or polyelectrolyte complexes (PECs) by forming intermolecular hydrogen bonds, electrostatic, hydrophobic and steric interactions in an aqueous solution [29]. The formation of these complexes depends on the molar mass, concentration and biopolymers ratio, in addition to the ionic strength, pH and temperature of the solution [30]. Natural polyelectrolytes, such as chitosan and alginates, contribute to the rheological–structural properties by forming a gelling network and changing their interfacial structure and stability [28].

Among the numerous native Brazilian foods, the fruits of assaí or açaí (*Euterpe oleracea*) stand out due to their diversity in nutritional and bioactive substances [31,32]. The fruits have been described as a “super fruit” due to its beneficial nutritional and health-related properties. The fruit pulp may contain ~50% lipid, mainly composed of monounsaturated (57 to 70%) and polyunsaturated (8 to 16%) fatty acids and up to 52% total carbohydrate; ~44% dietary fiber; ~8% protein, as well as other micronutrients [31,33,34]. More than 90 bioactive substances, such as flavonoids (31%), phenolic compounds (23%), lignoids (11%) and anthocyanins (9%), have been described in its oil and extracts [35,36,37]. In particular, açaí pulp oil has shown good antioxidant properties (around 2056.25 g of oil g^−1^ against DPPH, expressed as EC_50_) and up to 1252 ± 11 mg GAE kg^−1^ of phenolic substances, such as procyanidin trimers and dimers, syringic, valinnic and p-hydroxybenzoic acids [34,35].

In this context, the main purpose of this work was to prepare and characterize active chitosan packaging films that were plasticized with a deep eutectic solvent based on a mixture of glycerol and choline chloride and incorporated with up to 10% of açaí pulp oil microparticles for potential food applications. The physical–chemical, mechanical and antioxidant properties of the prepared films were characterized by different techniques. In our study, the results suggested that the chitosan films plasticized with 5% of DES and incorporated with 1–2% of açaí oil microparticles had the best combined mechanical and antioxidant properties as a potential material for food packaging.

## 2. Results and Discussion

### 2.1. Morphological Observations

**Figure 1:**
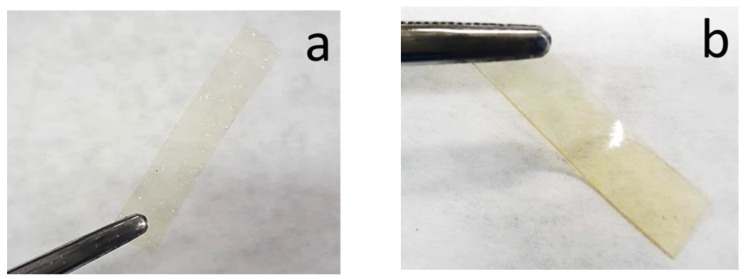
Photographs of rectangular sample films (**a**) F0/0 and (**b**) F5/0 films.

The solution casting method was used to produce chitosan composite films plasticized with a DES (1:2 ChCl:Gly) and incorporated with 0–10% of açaí PECs. The produced films had a yellow-gold transparent appearance without any visible opaque appearance or cracks (Figure 1). The unplasticized chitosan film, F0/0 (0% DES and 0% açaí PECs), was visually more brittle, transparent and thinner than the others, as expected [21]. All films were stored in a vacuum desiccator at room temperature for more than a year. After this time, the films were visually inspected, and found to be without signs of microbial contamination or deterioration, such as darkening or opaque aspects, cracks or frostbites (not shown), suggesting that it had good stability under the storage conditions used. The SEM and CLSM techniques were used to observe the morphology of the films and to confirm the presence of the açaí PECs.

**Figure 2:**
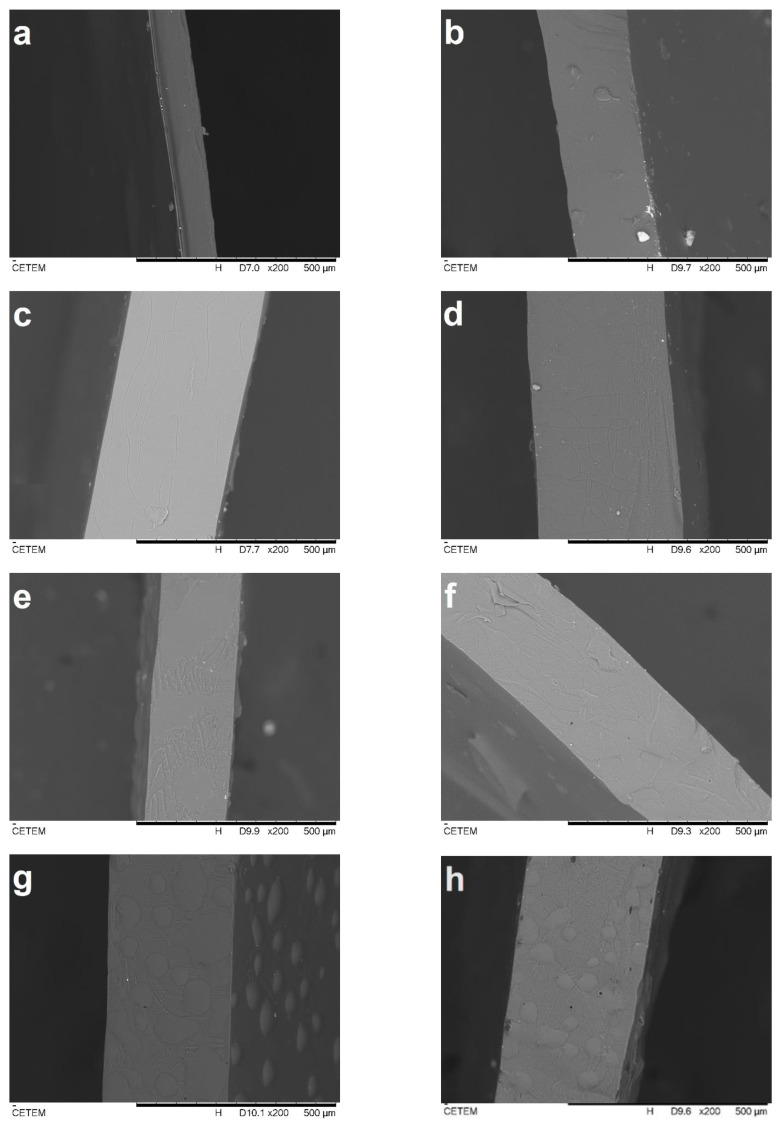
SEM observations of cross-sections of F0/0 (**a**), F5/0 (**b**), F5/0.25 (**c**), F5/0.5 (**d**), F5/1 (**e**), F5/2 (**f**), F5/5 (**g**) and F5/10 (**h**) films at 200× magnification.

The SEM cross-sections of the cryo-fractured films are presented in Figure 2. All films presented a dense, continuous and smooth surface without roughness, which could be related to the CS film-forming capability and miscibility between components [13,21,38]. It can be observed that the F0/0 sample, as pure chitosan control film (Figure 2a), was thinner than both the F5/0 films (Figure 2b) and the others (Figure 2c–h). This was expected since the addition of plasticizers to biopolymers increases the free volume and intermolecular spaces of their matrices [13]. The presence of the PECs with size < 370 µm [39] could have also contributed to this effect and were visualized as rounded shades in the cross-sections of the films F5/5 (Figure 2g) and F5/10 (Figure 2h).

All film samples other than those mentioned presented a dense, continuous and smooth surface (Figure 2b–f). This result is an indication of good miscibility between all components [21,38]. In these figures, it is also possible to observe larger particles that may have been formed during drying by coalescence or aggregation. Dammak et al. [40] observed a similar behavior in gelatin composite films incorporated with rutin microparticles. These authors revealed that the pure gelatin film (control) had a lower surface roughness, whereas films incorporated with the rutin microparticles presented a rougher surface and some flaws. Overall, the CS-DES-PECs films were dense and visually homogeneous.

**Figure 3:**
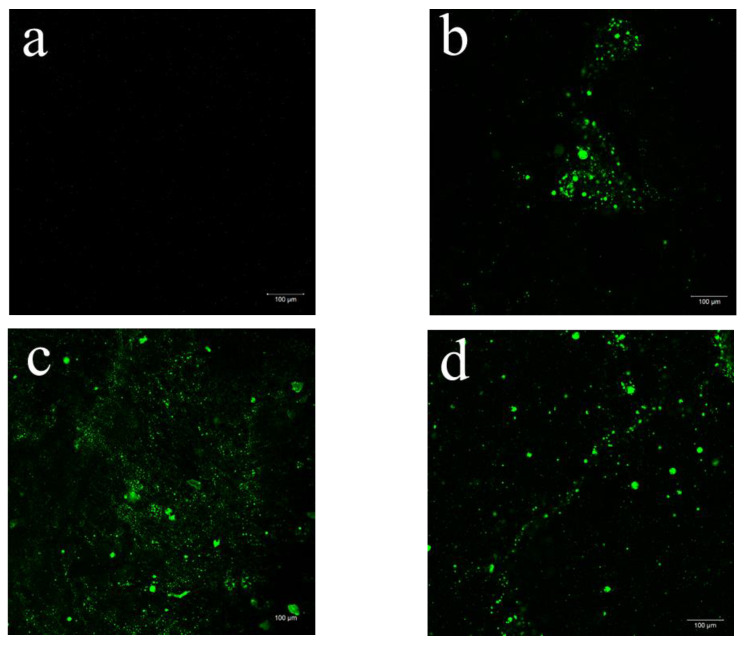

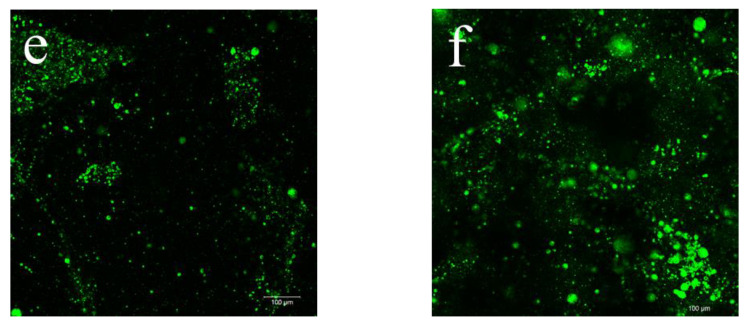
Selected 2D micrographs via CLSM of CS-DES films incorporating 0% (**a**), 0.25% (**b**), 0.5% (**c**), 2% (**d**), 5% (**e**) and 10% (**f**) of açaí PECs at 10× magnification. The encapsulated açaí oil within the PECs appears green while the CS-DES based films appear black.

CLSM allows deeper observation inside the bulk of films due to the fluorescent labelling [41]. This technique was used to evaluate the incorporation as well as the distribution of the açaí PECs inner the plasticized CS films. The CLSM examinations of the CS-composite films at 10× magnification are displayed in Figure 3.

It was possible to observe the Fluorescent Green dyed açaí PECs incorporated into the CS-composite films, which appeared in black (Figure 3). The dyed açaí oil within the microparticles was detected well by fluorescence [42]. Figure 3a shows the control CS-DES film (F5/0) at 10× magnification, which did not display any fluorescence and was used as a negative control with the other stained films. The frequency of fluorescent regions clearly increases with increasing proportion of PECs incorporated into the films, F5/0 (Figure 3a) < F5/10 (Figure 3f). A more meticulous observation at different focal planes revealed some PEC aggregation and a heterogenous distribution in the films with increasing amounts of the particles F5/0.25 (Figure 3b) > F5/10 (Figure 3f). This behavior could be the result of water evaporation during film drying, leading the particles to aggregate. Furthermore, it was possible to observe particles standing upon each other in different layers of the film. This behavior was also noted by Silva et al. [42] in chitosan/virgin coconut oil-based films.

Moreover, other magnifications at 20× and 40× were selected to better observe the particle morphology in the CS-composite films at higher levels of açaí PEC incorporation, as shown in Figure S1 (Supplementary Material). It was noted (Figure S1a–d) that the PECs exhibited spherical-like structures, similar to vacuoles containing the açaí oil (stained in green), which is a typical behavior of coacervates [43]. Similar observations were reported by Silva et al. [42] when studying the morphology of stained chitosan/virgin coconut oil-based emulsion films by CSLM. These authors found that, at lower oil concentration, the CS films exhibited a more homogenous distribution of the lipid droplets. Furthermore, they observed that, as the coconut oil concentration increased, the occurrence of aggregates or larger oily particles also increased, possibly because of changes in interfacial tension of the emulsion droplets during film drying.

### 2.2. Thickness and Water Vapor Permeability (WVP) of the Films

The WVP of biobased films is related to the exchange of moisture that occurs from the environment or the coated food through the films, considering its thickness and the differential pressure. The WVP can correlate the phenomenon of mass transfer, including the diffusion and solubility of water moieties, through the polymeric matrix. Since foods can absorb or lose water during their shelf-life, films and coatings must have the WVP determined prior to their utilization in packaging. This allows the mass transfer phenomena, and its affect on food quality, to be better controlled. Thickness and WVP values for the CS-composite films are presented in Table 1.

The WVP rises with the increase of PECs content in the films. The films without PECs presented the lowest WVP when compared to the others. The F0/0 film, which was the control, was formulated without DES and PECs and exhibited the lowest WVP at 1.1 × 10^−10^ g m^−1^ s^−1^ Pa^−1^ (*p* < 0.05). This result is similar to the value of 1.08 × 10^−10^ g m^−1^ s^−1^ Pa^−1^ found by Rivero et al. [13] for unplasticized chitosan films. These same authors found that the addition of 0.25% of plasticizer to CS films significantly increased their WVP. However, in the present work, the CS film plasticized with 5% of DES, sample F5/0, did not show a significant difference (*p* < 0.05) in WVP when compared to the F0/0 film. The WVP of the films incorporated with the PECs were significantly higher than the films without it. This may be explained by the increase in thickness and PECs content, which could modify the polymer network by decreasing the density or local viscosity and increasing the mobility of molecules that are diffusing through the films [40,44]. Furthermore, a reduction of film barrier properties may occur with the incorporation of vegetable oils [4]. Films based on polysaccharides and lipids are reported to display WVP values ranging from 0.7 × 10^−10^ g m^−1^ s^−1^ Pa^−1^ to 4.0 × 10^−10^ g m^−1^ s^−1^ Pa^−1^, which is the range of our findings [40].

### 2.3. FTIR Spectroscopy

**Figure 4:**
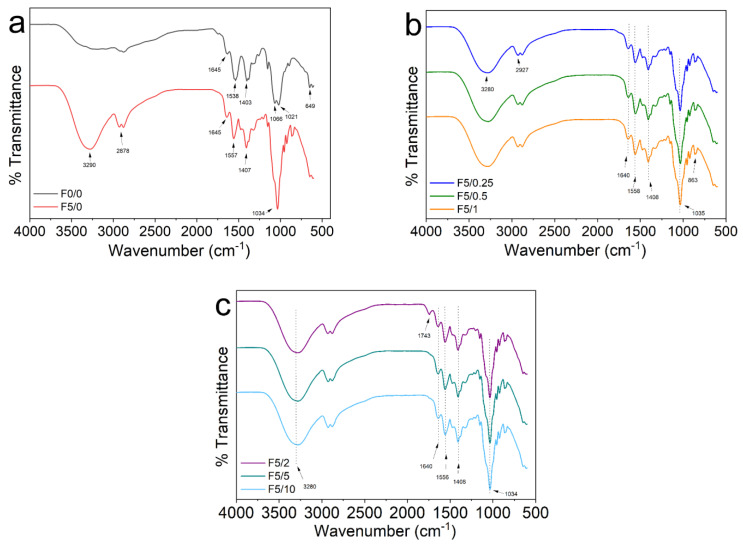
FTIR spectra for (**a**) pure CS (F0/0) and CS-DES (F5/0), (**b**) F5/0.25, F5/0.5 and F5/1, and (**c**) F5/2, F5/5 and F5/10.

Infrared spectroscopy was used to investigate the molecular structure of films as well as the interactions occurring between CS, DES and the açaí-incorporated PECs. The FTIR spectra of these materials are displayed in Figure 4. The spectrum for the F0/0 film presented high intensity bands associated with N−H bending (amide II), C−O symmetric stretching vibrations, C–O–C antisymmetric stretching, C−O stretching and C–H bending (rocking) vibrations, at 1538 cm^−1^, 1403 cm^−1^, 1066 cm^−1^, 1021 cm^−1^ and 649 cm^−1^, respectively (Figure 4a, black line) [21,45]. In the F5/0 film, it was noted that the addition of DES (Gly:ChCl) produced broad bands around 3290 cm^−1^ and 1034 cm^−1^ that were related to O−H and C−O stretching, respectively (Figure 4a, red line). These changes can be attributed to the formation of hydrogen bonds between CS and DES and also by the –O-H···Cl− bonds between Gly and ChCl in DES [46]. The FTIR spectrum for the CS-composite films displayed in Figure 4b,c are quite similar to the patterns of F5/0 film (Figure 4a). These results are in accordance with the existing literature [21,46]. It is worth noting that the bands attributed to amide I and amide II were changed from 1645 cm^−1^ and 1540 cm^−1^ to around 1640 cm^−1^ and 1558 cm^−1^. This result suggests an interaction between the components of the CS-composite films. This behavior was also observed by Pereira and Andrade [21] in CS-DES-curcumin films. In the F5/2 film, the low intensity band that appeared around 1743 cm^−1^ may be associated to the C=O stretching vibration of ester carbonyl functional groups either from the açaí oil or to the presence of DES. The association of this band to the açaí oil can be justified since it is frequently linked to triglycerides [47]. The presence of this band was also reported by Sokolova et al. [46] in chitosan films plasticized with of the mixture of malonic acid and ChCl. Likewise, Delgado-Mellado et al. [48] reported a band around 1715–1734 cm^−1^ in the FTIR spectra of different DES mixtures.

### 2.4. X-ray Diffraction (XRD)

**Figure 5:**
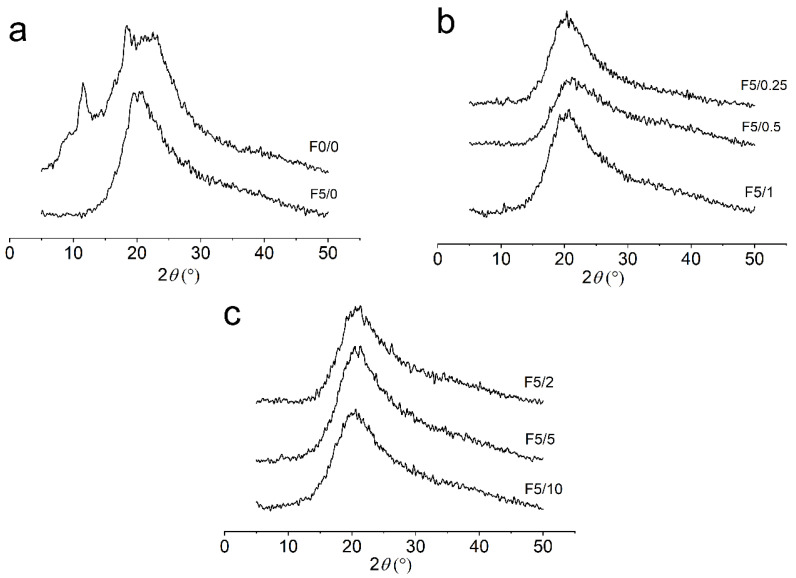
XRD patterns of the CS composite films for (**a**) pure CS (F0/0) and CS-DES (F5/0), (**b**) F5/0.25, F5/0.5 and F5/1, and (**c**) F5/2, F5/5 and F5/10.

The XRD analyses were performed to evaluate the changes in the amorphous and crystalline structures of the CS composite films (Figure 5). The diffractograms showed that the CS-composite films display a semicrystalline structure. The pure chitosan film, F0/0, showed three strong reflections around 2*θ* values of 11.5°, 18.4° and 23.3° (Figure 5). The peak around 11.5° confirms that CS films presented a semicrystalline structure, which may be related to the hydrated crystalline structure of CS caused by the integration of water molecules in its crystal lattice [49,50]. The peaks around 2*θ* values of 18.4° and 23.3° can be assigned to the crystal lattice and to the amorphous structure of CS, respectively [51]. This last peak was cited as being a typical fingerprint in chitosan films by Sun et al. [52]. XRD patterns for the CS films changed with the addition of DES.

The main characteristic observed in these diffractograms is the decrease in crystallinity in the plasticized CS films when compared to the unplasticized F0/0 ones. The peak at 2*θ* = 11.5° was significantly weakened with the addition of DES, as can be visualized in the diffractogram for the plasticized CS films (Figure 5). This result can be justified by the loss of hydrogen bonds between amino and hydroxyl groups in CS due to complexation with the plasticizer, which results in the increase in the amorphous structure of CS-DES films. Wong et al. [53] also observed that the addition of DES to chitosan–carboxymethylcellulose films significantly reduced the intensity of the reflection at 2*θ* = 2.9°. The F5/0 film exhibited only one broad peak around 2*θ* values of 20.4° (Figure 5a). This behavior was also observed in the other films, each of which displayed only one broad peak at 2*θ* = ~20°, as can be visualized in Figure 5b,c.

The crystallinity degree (CrD) of the films was estimated as the percentage ratio of crystalline and amorphous areas, and the results are listed in Table 2. The pure CS film displayed an estimated CrD close to 8%. This result is close to the value of ~7% determined for chitosan-based films by Silva et al. [42]. These authors also found a much higher value of CrD around 44% for chitosan powder. Escárcega-Galaz et al. [54] found that CS films at 2% concentration showed a degree of crystallinity around 9.2%, and that this value changed to 8% when glycerol was added.

In other works, CS-based films exhibited higher values of crystallinity degrees ranging from 37.8 to 46%. The DA values for the CS samples used in these cases were reported as 85 and 88%, respectively [55,56]. The estimated CrD decreased with the addition of plasticizer and the açaí PECs. The film F5/1 presented the lowest crystallinity degree at close to 4%. As the composition in plasticizer was kept constant at 5% in the CS-DES films, it is reasonable to suggest that the addition of açaí PECs influenced their CrD. Furthermore, it is worth noticing that the addition of 2, 5 and 10% (F5/2, F5/5 and F5/10) of açaí PECs in the films may have contributed to increasing the CrD to ~6%, although this is a lower value than the CrD of F0/0. This result corroborates the hypothesis that açaí PECs may interact with the CS matrix and result in a more organized structure and enhanced crystallinity. Moreover, this hypothesis is supported by the results of mechanical properties found for those same films, which displayed a lower elasticity as discussed in the following topics.

### 2.5. Mechanical Properties and DMA of the Films

**Figure 6:**
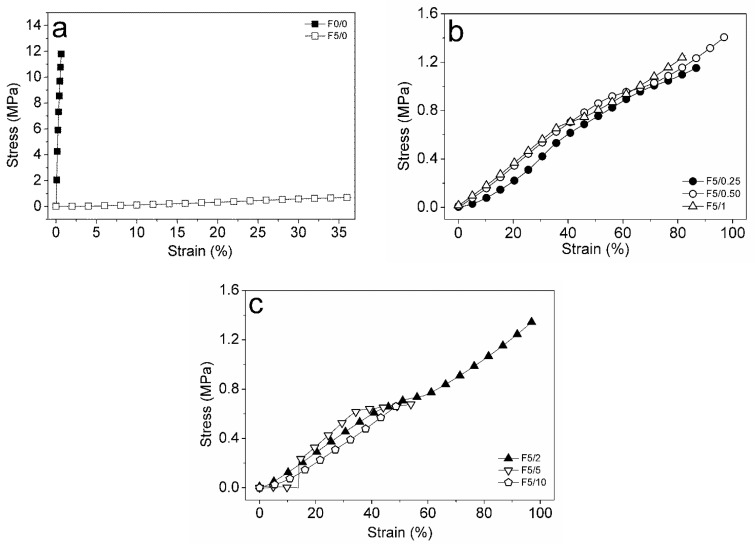
Stress versus strain curves for films: (**a**) F0/0 (full squares) and F5/0 (open squares); (**b**) F5/0.25 (full circles), F5/0.5 (circles), F5/1 (triangles); (**c**) F5/2 (full triangles), F5/5 (inverted triangles) and F5/10 (lozenges).

The tensile strength (TS or σ_max_), elongation at break (EB or ε_max_) and Young’s modulus (YM or Ε) for the CS-DES based films incorporated with açaí PECs at different ratios are shown in Table 2. The control film, F0/0, displayed a TS value around 11 MPa. Benbettaïeb et al. [57] reported a TS value for pure chitosan film of around 16 MPa. The addition of DES significantly affected the mechanical properties of the films when compared to the F0/0 and the F5/0 (Figure 6), as expected [13]. In the work of Pereira and Andrade [21], the addition of DES as plasticizer in chitosan-based films decreased the TS from ~24 to ~20 MPa; a significant decrease in TS was also obtained using glycerol. In general, the addition of DES as well as the açaí-PECs decreased TS when compared to the unplasticized control film, F0/0. Observing the plasticized CS-based films, it may be noted that the incorporation of açaí PECs had no significant effect on TS (*p* > 0.05).

The EB for the unplasticized film, F0/0, was around 0.6% and significantly increased to ~36% with the addition of DES, F5/0 (*p* < 0.05) (Figure 6a). The incorporation of açaí PECs positively affected EB, which reached the highest value around 104% for the F5/2 film (*p* < 0.05) (Figure 6b). It is also seen that the films exhibited a lower EB value above 2% PEC incorporation (Figure 6c). This effect can be related to a higher interference on the CS-DES continuous network by a higher amount of PECs. Above 2%, the addition of PECs seems to disrupt the matrix continuity and promote fragility. The YM of pure chitosan films was close to 3 × 10^3^ MPa, whereas the CS-DES films exhibited significantly lower values, less than 2 MPa (*p* < 0.05). Thus, the addition of DES produced less rigid films than pure chitosan. It was observed that, between the plasticized films, the YM decreased with the increase in PEC composition, although the differences were not significant (*p* > 0.05). This result is in accordance with the previously presented EB values, which suggested that CS-DES films incorporated with açaí PECs were more flexible than those of pure chitosan.

**Figure 7:**
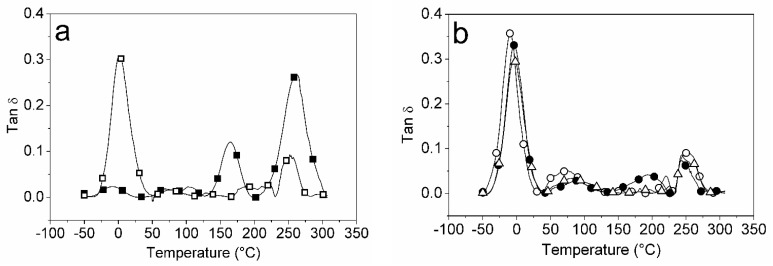
Variation of tan d with temperature for films: (**a**) F0/0 (full squares) and F5/0 (open squares); (**b**) F5/0.5 (open circles), F5/2 (full circles) and F5/10 (open triangles).

DMA was used to investigate the dynamic mechanical performance of pure CS and CS-DES composite films. The results are expressed by the loss (E″) and storage modulus (E′), or as the loss factor tan delta (tan δ) versus temperature, as shown in shown in Figure 7. The F0/0 film (pure CS film) presented three peaks attributed to the γ, β and α transitions, which are associated to conformational relaxations of the amorphous phase (Figure 7a). The first transition, γ, generally occurs at low temperature when the polymer in the glassy state and is associated to the relaxation of small side and terminal groups. It is possible to observe that the DES addition promoted a shift in the γ transition from −8 °C in F0/0 (pure CS) to 2 °C in the F5/0 films, which is associated with the plasticizing effect of DES. The β transition temperature for the F0/0 film was observed around 165 °C, whereas this peak weakened and broadened in the CS-DES films. This could be associated with the interactions of CS and DES, which suppressed the ordering of CS molecular chains and reduced their crystallinity. This effect was also observed by Tuhin et al. [58] in the CS-starch-Gly-mustard oil films. The maximum temperature of the peak of tan δ in the F0/0 film was close to 263 °C, whereas it ranged from −9.2 to 2.2 °C in the plasticized CS-composite films (Figure 7b). This result confirms the plasticizing effect of the DES on the CS-composite films. The tan δ and E′ values for the films at 25 °C are listed in Table S1 (Supplementary Material), where a marked decrease in E′ on the addition of DES to the CS films may be noted, indicating promotion of relaxation of the CS chains by the plasticizer. This is in accordance with the XRD findings, which showed a decrease in CS crystallinity with the addition of DES to the films. Pereira and Andrade [21] also found three peaks of tan δ at −1.5 °C, 73.9 °C and 166.8 °C for pure CS film.

### 2.6. Thermogravimetric Analysis (TGA) of the Films

**Figure 8:**
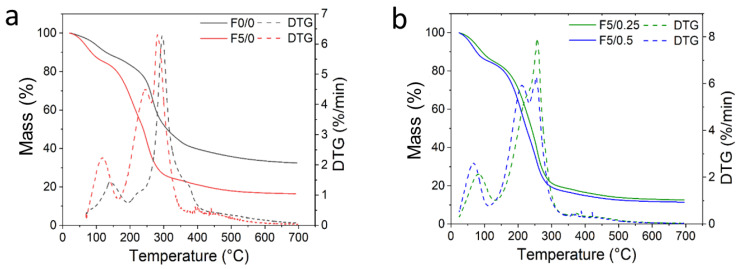

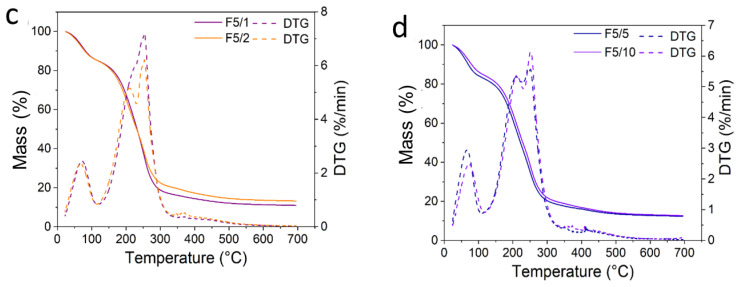
TGA (continuous lines) and DTG (dashed lines) curves for (**a**) CS-based films, F0/0–F5/0; (**b**) F5/0.25–F5/0.5; (**c**) F5/1–F5/2; and (**d**) F5/5–F5/10.

The TGA and its derivative (DTG) curves were used to evaluate the thermal stability of the CS-DES composite films, shown in Figure 8. The TGA and DTG data, initial temperature of degradation (T*_onset_*), maximum temperature of degradation (T*_peak_*) and percentage of residue at 700 °C are listed in Table S2 (Supplementary Material).

It is observed that the F0/0 film presented a thermal degradation behavior occurring in two steps (Figure 8a). The first step of mass loss ranged from around 100 to 200 °C, attributed to water evaporation [46,59]. The second mass loss ranged from around 200 to 400 °C, which is correlated with the thermal degradation of chitosan through the dehydration of glycosidic rings and its chain depolymerization [21]. T*_peak_* of F0/0 was close to ~290 °C and ~300 °C, as observed by Kamdem et al. [59] and Sokolova et al. [46], respectively, for pure CS films. It is noteworthy that the Tonset in the plasticized CS film, F5/0, occurred at a lower temperature due to the influence of DES on the CS chain mobility, resulting in a decreased thermal stability (Figure 8a). This plasticizing behavior reflects on T*_onset_* of the CS-composite films, which decreased from ~240 °C in F0/0 to ~170 °C in F5/0 film. Observing the DTG curve for the F5/0 film, a nonresolved peak may be observed at 209.2 °C, together with the main peak at 246.7 °C. These peaks were attributed to the maximum degradation of plasticizer and chitosan, respectively. A similar result was also observed by Sokolova et al. [46] for CS films plasticized with different proportions of DES (malonic acid:ChCl). The thermal degradation behavior for CS-DES films occurred mainly in two steps, as can be visualized in Figure 8b–d. The decreasing thermal stability of the CS composite films incorporated with açaí PECs was also observed. For them, the second event occurred at Tonset and ranged from 201 °C to 182 °C in F5/0.25 and F5/10 films, respectively (Figure 8b–d). This result suggests that the CS-composite films degraded at higher temperatures than the F5/0 film, indicating that the presence of açaí oil microcapsules improved thermal stability since the DES content was kept constant. The T*_peak_* in the composite films was slightly different, ranging from 256 °C to ~251 °C.

### 2.7. Antioxidant Properties

**Figure 9:**
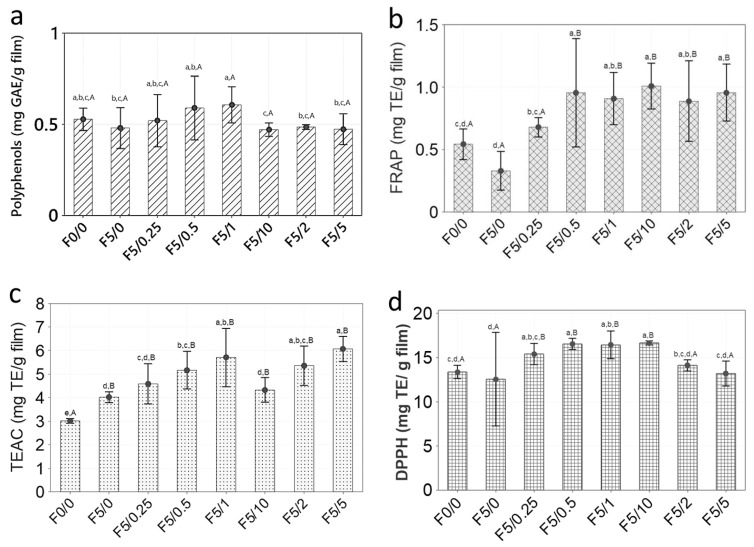
Antioxidant properties of CS-based films determined by TPC (**a**), FRAP (**b**), TEAC (**c**) and DPPH (**d**) assays. Different lowercase (a, b, c, d) or uppercase (A, B) letters within a column indicate significant differences between samples by Tukey’s test or Dunnett’s test, respectively (*p* < 0.05).

The in vitro total polyphenols content (TPC) and antioxidant activity of CS-composite films is presented in Figure 9 and are expressed as mean (column) ± standard deviation (bar) (n = 3). For comparison purposes, the antioxidant properties of the açaí pulp oil were evaluated using the same techniques.

The results of TPC were calculated using a linear regression (Equation (1)) that used a standard gallic acid curve, expressed as mg of gallic acid equivalent (GAE) per g^−1^ of film.

(1) $$TPC=\frac{Absorbance_{sample}-0.0696}{0.0031}\;R^{2}=0.9988$$

**Figure 10:**
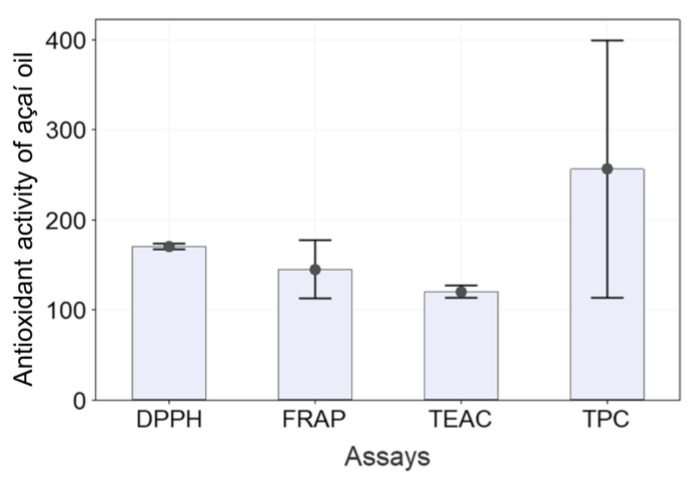
Antioxidant activity of açaí oil by DPPH radical scavenging, FRAP, TEAC and TPC assays. The results shown are mean (column) ± standard deviation (bar) (n = 3) values for DPPH, FRAP and TEAC assays, expressed as mg TE L^−1^ of oil, and for TPC as mg GAE L^−1^ of oil.

The CS-based films exhibited a low value of TPC, ranging from 0.47 to 0.61 mg of GAE g^−1^ of film. It was noted that the F5/1 film presented the highest TPC value, followed by the F5/0.5 film. However, no significant statistical difference was observed among all films, either by the Tukey or Dunnett’s test (*p* > 0.05), as can be observed in Figure 9. Likewise, this result reflects the effect of microencapsulation of açaí oil in PECs on preserving its bioactivity, allowing them to be released when in contact with a food surface. Moreover, it has been reported that CS films display a low value of TPC, <2 mg GAE g^−1^ [60,61,62]. This mild scavenging property is due to the interaction of free radicals with the residual free amino groups (−NH_2_) from CS forming ammonium groups (−NH_3_^+^) [62]. The açaí oil exhibited an antioxidant activity determined by TPC of 256.05 ± 57.45 mg GAE L^−1^, as shown in Figure 10. Pacheco-Palencia et al. [35] found that the açaí oil had a total soluble phenolic content around 1252 ± 11 mg GAE kg^−1^. These authors found high content of phenolic substances, such as procyanidin trimers and dimers, syringic, valinnic and p-hydroxybenzoic acids in the açaí pulp oil and suggested that some phenolic substances could be deposited as free or bound compounds onto the nonpolar lipid phase, thus giving it high antioxidant activity.

The FRAP of the CS-based films was determined using a standard curve of Trolox, calculated using a linear regression (Equation (2)) and expressed as mg of Trolox equivalent per g of *sample*.

(2) $$FRAP\;\left({\mathrm{mg}\;\mathrm{TE}\;g}^{-1}\right)=\frac{Absorbance_{sample}-1.7515}{0.0033}\;R^{2}=0.9979$$

The antioxidant power of CS-based films ranged from ~0.5 to 1.0 mg TE g^−1^ of film. The control *sample*, F0/0, presented an antioxidant value around 0.5 mg TE g^−1^ of film, whereas the F5/10 presented a value two times higher. These results are in accordance with the studies of Ruiz-Navajas et al. [60]. These authors found an antioxidant power by FRAP of 0.21 mg TE g^−1^ for pure CS film. It was also observed that the antioxidant power of the films significantly increased with the increase in incorporated açaí PECs (*p* < 0.05). When taking the F0/0 film as control, the films F5/0.5, F5/1, F5/2, F5/5 and F5/10 were statistically different (*p* < 0.05 by Dunnett’s test). Cuevas-Acuña et al. [63] studied the antioxidant properties of chitosan–gelatin composite films by FRAP and found values ranging from ~300 to 600 µmol TE mg^−1^ of film. In another study, conjugated chitosan grafted with stearic acid and gallic acid exhibited an antioxidant activity by FRAP assay, expressed as IC50, ranging from ~141 to 367 µg mL^−1^ [64]. The antioxidant power of açaí oil by FRAP was 144.95 ± 13.02 mg TE g^−1^, as shown in Figure 10.

The TEAC for the CS-based films using the ABTS radical reagent was calculated using a liner regression (Equation (3)) and expressed as mg of TE g^−1^ of film.

(3) $$TEAC\;\left({\mathrm{mg}\;\mathrm{TE}\;g}^{-1}\right)=\frac{Absorbance_{sample}+0.6783}{0.0019}\;R^{2}=0.9912$$

The antioxidant activity for the CS-based films ranged from around 3 to 6 mg TE g^−1^ of film. The CS-composite films incorporated with açaí PECs were almost two times higher than the control, F0/0, exhibiting a significant difference (*p* < 0.05 Dunnett’s test). The film F5/5 exhibited the highest antioxidant capacity, followed by F5/1 and F5/2. Thus, it is possible to suggest that the açaí PECs contributed to an increase in the antioxidant activity of the CS-composite films. The açaí oil had an antioxidant capacity against the ABTS radical around 120.21 ± 2.77 mg TE g^−1^, as shown in Figure 10. Cuevas-Acuña et al. [63] studied the antioxidant properties of chitosan–gelatin composite films by ABTS radical scavenging assay and found values ranging from ~200 to 900 µmol TE mg^−1^ of film. In another work, CS films incorporated with mango leaf extract presented an antioxidant capacity by ABTS radical scavenging assay ranging from ~2 to 8 µg GAE g^−1^ film [65]. CS/gelatin composite films incorporated with gallic acid presented an antioxidant capacity against ABTS radical ranging from 20 to 60% [66].

In this work, the antioxidant capacity against the DPPH radical was calculated using a liner regression (Equation (4)), and the results were expressed as mg of TE g^−1^ of film.

(4) $$DPPH\cdot \left({\mathrm{mg}\;\mathrm{TE}\;g}^{-1}\right)=\frac{Absorbance_{sample}-0.3622}{0.0011}\;R^{2}=0.9991\;$$

The CS-based films displayed an antioxidant activity against the DPPH radical ranging from around 13 to 17 mg TE g^−1^ of film. These values were higher than for pure CS film, 0.003 mg TE g^−1^, as reported by Ruiz-Navajas et al. [60]. Additionally, it was noted that the CS-composite films with higher açaí PECs exhibited the highest antioxidant activity against DPPH. The F5/10 film had significantly higher antioxidant capacity than the control, F0/0 (*p* < 0.05 by Tukey and Dunnett’s test). In another work, Moradi et al. [67] found that CS films displayed an antioxidant capacity against DPPH radical of around 10%. Rui et al. [66] found that the CS film presented a DPPH radical antioxidant capacity around 15% and by ABTS assay, around 20%. Riaz et al. [68] determined the antioxidant activity of CS-based films with apple peel polyphenols by DPPH and ABTS radical scavenging ranging from 20 to 95%. Other work with CS–apple polyphenols films found values of DPPH radical scavenging activity ranging from 35 to 95% [52]. In the work of Siripatrawan and Vitchayakitti [62] the antioxidant activity via DPPH assay for CS–propolis films exhibited values ranging from around 5 to 55%. The açaí oil studied in this work displayed an antioxidant activity against the DPPH radical of 170.3 ± 1.3 mg TE L^−1^ oil, as shown in Figure 10. Another study by Rufino et al. [34] found that açaí oil has an antioxidant activity around 2056.25 g of oil g^−1^ against DPPH, expressed as EC_50_. Thus, the antioxidant activity determined for the CS-composite films incorporated with açaí PECs may have contributed to the bioactivity of films.

## 3. Materials and Methods

### 3.1. Materials

Medium molar mass chitosan (CS) was purchased from Sigma Aldrich Brazil Ltd. (São Paulo, SP, Brazil). Its degree of acetylation (DA = 15%) was determined by ^1^H NMR spectrometry in D_2_O/DCl at 65 °C. Its viscosity average molar mass (${\bar{M}}_{v})$ of ~2.97 × 10^5^ was determined previously [69]. Glycerol (Gly) and choline chloride (ChCl) were purchased from Vetec Química Fina Ltda. (Rio de Janeiro, RJ, Brazil) and Sigma Aldrich Brazil Ltd. (São Paulo, SP, Brazil), respectively. Açaí pulp oil (AO) was purchased from Beraca Ingredientes Naturais S.A. (Ananindeua, PA, Brazil). Folin-Ciocalteau’s phenol reagent, 2,2′-azino-bis-3-ethylbenzothiazoline-6-sulfonic acid (ABTS), 2,2-diphenyl-1-picrylhydrazyl (DPPH), gallic acid and (±)-6-hydroxy-2,5,7,8-tetramethyl-chroman-2-carboxylic acid (Trolox) as well as 2,4,6-Tris(2-pyridyl)-s-triazine (TPTZ) were purchased from Sigma–Aldrich (Milan, Italy). All other chemicals used in this study were of analytical grade. Ultrapure water from a Millipore Direct-Q 3 UV system was used.

### 3.2. Preparation of the Deep Eutectic Solvent and the CS-Composite Films

The deep eutectic solvent (DES) was prepared by mixing the ChCl powder (hydrogen bond acceptor) with Gly (hydrogen bond donor) at the molar ratio of 1:2 (*w*/*w*) [70], under constant magnetic stirring at 80 °C to obtain a homogenous mixture. The DES mixture was left to cool down and stored at room temperature until further analysis.

To film preparation, CS powder at 2.5% (*w*/*v*) was dissolved in a 1% (*v*/*v*) acetic acid solution under constant magnetic stirring overnight at room temperature. Later, the DES solution was added at 0 and 5% (*v*/*v*), as listed in Table 1, and then stirred overnight at 25 °C until full homogenization. The optimized açaí PECs (8:2 chitosan: sodium alginate PECs incorporated with 0.5% of açaí oil and cross-linked with 0.5% of CaCl_2_) were prepared according to our previous work [39] and incorporated at different concentrations from 0 to 10% (*v*/*v*) into the CS-DES solution to obtain the film-forming mixture.

**Scheme 1:**
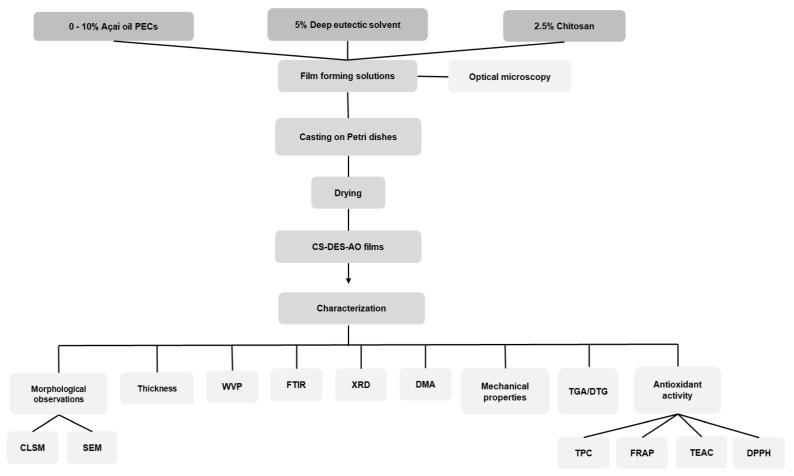
Flowchart of the experimental process to obtain and characterize the chitosan/deep eutectic solvent films containing açaí–filled microcapsules.

The film-forming solutions were cast in Petri dishes and dried in an oven at 60 °C for 24 h, followed by drying at room temperature in a desiccator under vacuum containing a modified atmosphere of anhydrous calcium chloride, ~25% of relative humidity (RH), until constant weight (~5 days), which resulted in the formation of thin CS-DES-PECs films (Scheme 1). The thickness of the films was controlled by casting the same weight (~15 g) of film-forming solutions in each plate. Finally, the films were stripped off from the glass plates, stored at room temperature and modified atmosphere (~25% of RH) until further analysis.

### 3.3. Morphological Observations of the Films

The morphological aspects of the composite films were observed by scanning electron microscopy (SEM) and confocal laser scanning microscopy (CLSM). For the SEM observations, cryofractured surfaces of the films were examined at 5 kV and photographed (TM3030 Plus, Hitachi, Tokyo, Japan). The CLSM assessments were performed according to Shi et al. [38] with some modifications, using a LSM880 Airyscan Zeiss confocal microscope (Zeiss, Oberkochen, German). The fluorescent dye Nile Red was used to dye the hydrophobic phase (açaí oil) of the PECs dispersed in the CS-DES films. The samples (~20 × 20 mm) were placed on concave microscope glass slides, stained with 2 drops of a 1 mg mL^−1^ Nile Red solution in dimethyl sulfoxide (DMSO), left to stain for approximately 3 min and covered with glass coverslips. To observe the presence of the açaí PECS loaded in the CS-DES-PECs films, the fluorescence of Nile Red was examined by exciting an argon laser at 488 nm and the applied magnification varied from 10 to 63×. For optimum image resolution the microscope pinhole was set up to 1 Airy unit (AU) and the range indicator tool on the Zen Image Software (Zeiss, Oberkochen, German) was used to adjust the proper red pixels saturation.

### 3.4. Thickness and Water Vapor Permeability of the Films

The film thicknesses (L) [mm] were measured with a digital micrometer model 13101-25 with a 0.001 mm resolution (PANTEC^®^, Rio Grande do Sul, Brazil). Five random measurements were taken on each testing sample at different points. For the determination of the tensile strength of the films, the thickness was measured along the length of each film strips. The results were expressed as mean ± standard deviation (SD).

The water vapor permeability (WVP) of the films was determined gravimetrically based on the E96/E96M assay from the Standard Test Method for Water Vapor Transmission of Materials [71]. Films with no visual physical defects, such as bubbles or cracks, were chosen for the WVP tests. Samples squares of ~20 × 20 mm were tightly sealed to the top of a plastic permeation cell with a pinhole of ~6 mm of diameter. The permeation cell contained distilled water (100% of RH) at a 3/4 level from the film. Paraffin was used to seal the films in the cells and to make sure that the water vapor permeation only diffused through the films. The permeation cells were weighed, placed in a desiccator containing granular anhydrous calcium chloride (~25% of RH) at room temperature and vacuum atmosphere was applied, providing a relative humidity gradient through the films. The cells were periodically weighed to evaluate the weight changes every 24 h for 14 days. The tests were carried out in duplicate for each film sample and the WVP in g m^−1^ s^−1^ Pa^−1^ was obtained from Equation (5).

(5) $$\mathrm{WVP}=\left(\Delta m\times L\right)/\left(A\times \Delta t\times \Delta p\right)$$

where Δm is the weight change (g), L is the film thickness (m), A is the area (0.006 m^2^) of the film exposed to water vapor permeation for a time Δt(s) to a partial water pressure Δp (Pa).

### 3.5. Fourier Transform Infrared (FTIR) Spectroscopy and X-ray Diffraction (XRD) of the Films

FTIR analyses of the composite films were carried out using a Frontier 98737 model spectrometer (Perkin Elmer, Waltham, MA, USA) at room temperature that was equipped with a Universal Attenuated Total Reflectance (ATR) cell device in the 4000–400 cm^−1^ range. All spectra were registered by averaging 60 scans with a resolution of 4 cm^−1^ in transmission mode.

The XRD analysis of the films were performed using the Ultima IV X-ray diffractometer (Rigaku Corporation, Tokyo, Japan) in continuous scanning mode with CuKα radiation generated at 40 kV and 20 mA. The data were collected over the angular region of 2° to 50° (2*θ*) at 0.05° per second (2*θ*). The crystallinity degree (CrD) of the films was calculated as the ratio of the crystalline area to the total area under diffraction peaks (peak deconvolutions were executed with Fytik^®^, an open-source software).

### 3.6. Mechanical Properties and Dynamic Mechanical Analyses (DMA) of the Films

Compression molded samples with dimensions of 14.0 mm × 8.0 mm × 0.3 were analyzed in a Q800 DMA (TA Instruments, New Castle, DE, USA) using a film clamp at 1 Hz under multi frequency-strain mode from −50 to 310 °C and at a heating rate of 3 °C min^−1^ [72]. A total of two measurements were taken for each sample. The results were presented as loss factor (tan δ) and given by the ratio between the loss modulus (E″) and the storage modulus (E′) as a function of temperature.

Tensile tests were carried out at 21 °C in a Q-800 DMA equipment (TA Instruments) according to the ISO 527-3 method and with rectangular compression-molded films 14.0 mm × 8.0 mm × 0.3 mm that had previously been conditioned at 21 °C and 50% relative humidity for a period of at least 48 h. The samples were placed between a fixed and a moveable film clamp, and the experiments were performed in triplicate in tensile mode. The tensile strength (σ), Young’s modulus (E) and elongation at break (ε) were determined [73].

### 3.7. Thermogravimetric Analysis (TGA) of the Films

TG analyses were performed in a Q500 TGA equipment (TA Instruments) under nitrogen flow. Samples weighing approximately 10 mg were heated from 25 to 700 °C at a heating rate of 10 °C min^−1^.

### 3.8. Antioxidant Properties of Açaí Oil and the Films

The antioxidant properties of the CS-DES-based films incorporated with the açaí PECs were determined by different in vitro antioxidant tests: Folin–Ciocalteu, Trolox equivalent antioxidant capacity (TEAC), DPPH radical scavenging and ferric reducing antioxidant potential (FRAP) assays. For all antioxidant analyses, a film extract was obtained by adding ~10 mg of film samples in Eppendorf vials containing 1 mL of 60:40 ethanol: distilled water solution, vigorously mixed for 1 min and then centrifuged. An aliquot of this supernatant solution was then combined in a 96-well plate with the appropriate reagents. For comparison purpose, the antioxidant properties of the açaí pulp oil were also evaluated by the same methods. To complete this, about 10 µg of the oils was added to 1 mL of ethanol, vigorously mixed for 1 min and centrifuged for 5 min in a mini-centrifuge (C1008-B-E MyFuge, Benchmark Scientific, Sayreville, NJ, USA) and the assays were performed as described in the following sections.

The Folin–Ciocalteu assay has been widely used to investigate the total phenolic content (TPC) in food matrixes, however it can also be applied to study antioxidants because its mechanism of action is based on an oxidation/reduction reaction [74]. This analysis was performed based on the methodology adapted from Singleton et al. [75]. A 10 μL sample of the supernatant solution (10 mg mL^−1^) was combined in a 96-well plate with 40 μL of Folin–Ciocalteu reagent and 150 μL of 4% sodium carbonate solution. The 96-well plate was incubated for 30 min at room temperature in the dark and the absorbance of film samples and a standard curve of gallic acid (0–500 µg mL^−1^) was read at 765 nm in a plate-reader spectrophotometer (Spark 10M, Tecan Trading AG, Switzerland). Distilled water was considered as blank. The samples were analyzed in triplicate and the results were compared with a gallic acid standard curve and expressed as µg GAE mL^−1^.

The DPPH radical scavenging method was employed based on the methodology adapted from Brand-Williams et al. [76]. The supernatant solution at 10 mg mL^−1^ concentration was mixed in a 96-well plate with 300 µL of the DPPH reagent and then incubated at 25 °C for 30 to 120 min in a dark room. The absorbances at 517 nm of the film extracts and the Trolox standard curve were read in a plate-reader spectrophotometer (Spark 10M, Tecan Trading AG, Männedorf, Switzerland). Distilled water was considered as blank. The samples were analyzed in triplicate and the results were compared with a Trolox standard curve (0–1000 µM) and expressed as mg TE L^−1^.

The Trolox equivalent antioxidant capacity (TEAC) assay was performed according to the methodology from Re et al. [77]. A 10 µL of the film extract was mixed in a 96-well plate with 300 µL ABTS solution. The absorbance of the film extracts and the Trolox standard curve was read after 6 min at 734 nm using a plate-reader spectrophotometer (Spark 10M, Tecan Trading AG). The samples were analyzed in triplicate and the results were compared with a Trolox standard curve (0–1000 µM) and expressed as mg TE L^−1^.

The ferric reducing antioxidant power (FRAP) assay was used according to the methodology adapted from Lotito and Frei [78]. In this procedure, 10 µL of the film extracts (10 mg·mL^−1^) were merged with 300 µL of FRAP reagent (containing a 10:1:1 mixture of 300 mM sodium acetate pH 3.6: 0.8 mM TPTZ: 1.7 mM FeCl3) in a 96-well plate and incubated for 15 min at 37 °C. The absorbance of the film extracts and the Trolox standard curve (0–1000 µM) were read at 593 nm in a plate-reader spectrophotometer (Spark 10M, Tecan Trading AG). The samples were analyzed in triplicate, and the results were compared with a Trolox standard curve and expressed as mg TE L^−1^.

### 3.9. Statistical Analysis

The results from WVP, mechanical properties and antioxidant assays were statistically analyzed using the Minitab^®^ version 19.2020.1 (Minitab, LLC, State College, PA, USA), by analysis of variance (ANOVA) and Tukey’s test at a significance level of 95% (*p* < 0.05). The FTIR spectra, X-ray diffraction patterns, variation of tan delta versus temperature curves and TGA curves were graphically analyzed using the OriginPro^®^ software version 2021 (OriginLab Corporation, Northampton, MA, USA).

## 4. Conclusions

This study showed that chitosan films plasticized with DES and incorporated with açaí microparticles had good physicochemical, mechanical and antioxidant properties. The CS-composite films had a dense visual and homogeneous appearance, indicating good miscibility between the components. Thickness, water vapor permeability, crystallinity, dynamic and mechanical behavior of the films, as well as their antioxidant properties, were influenced by the incorporation of the açaí microparticles. The mechanical behavior, especially the tensile strength and the elongation at break were significantly affected by the addition of DES and increased açaí microparticle inclusion. The highest elongation at break (ε*_max_*), close to 104%, was noted for the films containing 2% of açaí microparticles. The highest antioxidant properties against the ABTS and DPPH radicals, up to 6 and 17 mg TE g^−1^, respectively, were observed for films with 1% of açaí microparticles, although this was not significantly different from the films with 2% of particles. Thus, these results suggest that chitosan–DES films incorporated with 2% of açaí PECs have the greatly improved mechanical and antioxidant properties and therefore significant potential as food packaging materials.

## Figures and Tables

**Table 1:** Formulation of the CS-DES based films incorporated with açaí PECs and their thickness and water vapor permeability.

Sample	DES (%)	Açaí PECs (%)	Thickness (mm)	WVP (g m^−1^ s^−1^ Pa^−1^)
F0/0	0	0	0.16 ± 0.02 ^c^	1.1 × 10^−10^ ± 1.0 × 10^−11 c^
F5/0	5	0	0.19 ± 0.03 ^bc^	1.9 × 10^−10^ ± 9.9 × 10^−12 bc^
F5/0.25	5	0.25	0.36 ± 0.02 ^a^	3.9 × 10^−10^ ± 3.2 × 10^−11 a^
F5/0.5	5	0.50	0.34 ± 0.01 ^a^	3.4 × 10^−10^ ± 2.3 × 10^−11 ab^
F5/1	5	1	0.33 ± 0.01 ^a^	3.9 × 10^−10^ ± 5.3 × 10^−11 a^
F5/2	5	2	0.27 ± 0.05 ^abc^	4.2 × 10^−10^ ± 9.9 × 10^−11 a^
F5/5	5	5	0.28 ± 0.04 ^abc^	2.9 × 10^−10^ ± 5.0 × 10^−12 ab^
F5/10	5	10	0.29 ± 0.06 ^ab^	3.4 × 10^−10^ ± 4.0 × 10^−11 ab^

Results are expressed as mean ± standard deviation. Different superscript letter within a column indicates a significant difference among samples by Tukey’s test (*p* < 0.05).

**Table 2:** Estimated crystallinity degree (CrD), tensile strength (TS), elongation at break (ε_max_) and Young’s modulus (YM) of CS-DES based films.

Sample	CrD (%)	TS (MPa)	ε_max_ (%)	YM (MPa)
F0/0	7.95	11.0 ± 1.5 ^a^	0.60 ± 0.1 ^d^	2927 ± 154 ^a^
F5/0	4.35	0.74 ± 0.07 ^b^	36.4 ± 1.9 ^c^	1.7 ± 0.1 ^b^
F5/0.25	4.45	1.20 ± 0.04 ^b^	89.2 ± 3.7 ^a^	1.7 ± 0.4 ^b^
F5/0.5	4.13	1.26 ± 0.16 ^b^	99.3 ± 4.5 ^a^	1.6 ± 0.4 ^b^
F5/1	3.93	1.13 ± 0.24 ^b^	83.0 ± 9.6 ^a^	1.7 ± 0.2 ^b^
F5/2	4.94	1.47 ± 0.31 ^b^	103.6 ± 8.4 ^a^	1.7 ± 0.2 ^b^
F5/5	5.23	0.63 ± 0.16 ^b^	52.0 ± 7.1 ^b^	1.4 ± 0.3 ^b^
F5/10	5.74	0.66 ± 0.08 ^b^	49.3 ± 2.3 ^b,c^	1.35 ± 0.06 ^b^

Results are expressed as mean ± standard deviation (n = 3). Different superscript letter within a column indicates a significant difference among samples by Tukey’s test (*p* < 0.05).

## Data Availability

Data sharing is not applicable to this article.

## Supplementary Materials

The following supporting information can be downloaded at: https://www.mdpi.com/article/10.3390/molecules28031507/s1, Figure S1: Selected 2D micrographs obtained by CSLM for CS-DES films, (a) F5/2 at 20×, (b) F5/2 at 40×, (c) F5/5 at 20×, and (d) F5/10 at 40× magnifications; Table S1: E′ (MPa) at 25 °C and tan δ (°C) for the CS-based films; Table S2: TGA and DTG data for CS-based films.
