# Supplementary material for: Improved Antioxidant and Mechanical Properties of Food Packaging Films Based on Chitosan/Deep Eutectic Solvent, Containing Açaí-Filled Microcapsules
Source: Molecules. 2023 Feb 3;28(3):1507. doi: 10.3390/molecules28031507 (PMC9920262; doi:10.3390/molecules28031507)
Supplement: Supplementary file 1 [file molecules-28-01507-s001.zip › molecules-2184367-supplementary.pdf]

molecules

Supplementary materials

for

Improved antioxidant and mechanical properties of food packaging films based on chitosan/deep eutectic solvent, containing açaí-filled microcapsules

Barbara E. Teixeira-Costa[1,2*], Willian H. Ferreira[3], Francisco M. Goycoolea[4], Brent S. Murray[5], Cristina T. Andrade[6]

[1] Faculdade de Ciências Agrárias, Universidade Federal do Amazonas, Avenida General Rodrigo Otávio 6200, Manaus 69077-000, AM, Brazil, betcosta@ufam.edu.br
[2] Programa de Pós-Graduação em Ciência de Alimentos, Instituto de Química, Universidade Federal do Rio de Janeiro, Avenida Moniz Aragão 360, Bloco 8G/CT2, Rio de Janeiro 21941-594, RJ, Brazil, betcosta@gmail.com.
[3] Programa de Pós-Graduação em Ciência de Alimentos, Instituto de Química, Universidade Federal do Rio de Janeiro, Avenida Moniz Aragão 360, Bloco 8G/CT2, Rio de Janeiro 21941-594, RJ, Brazil, whermogenes@gmail.com.
[4] School of Food Science and Nutrition, University of Leeds, LS2 9JT Leeds, UK, F.M.Goycoolea@leeds.ac.uk.
[5] School of Food Science and Nutrition, University of Leeds, LS2 9JT Leeds, UK, B.S.Murray@food.leeds.ac.uk.
[6] Programa de Pós-Graduação em Ciência de Alimentos, Instituto de Química, Universidade Federal do Rio de Janeiro, Avenida Moniz Aragão 360, Bloco 8G/CT2, Rio de Janeiro 21941-594, RJ, Brazil, ctandrade@iq.ufrj.br.

*Correspondence: betcosta@ufam.edu.br

molecules MDPI

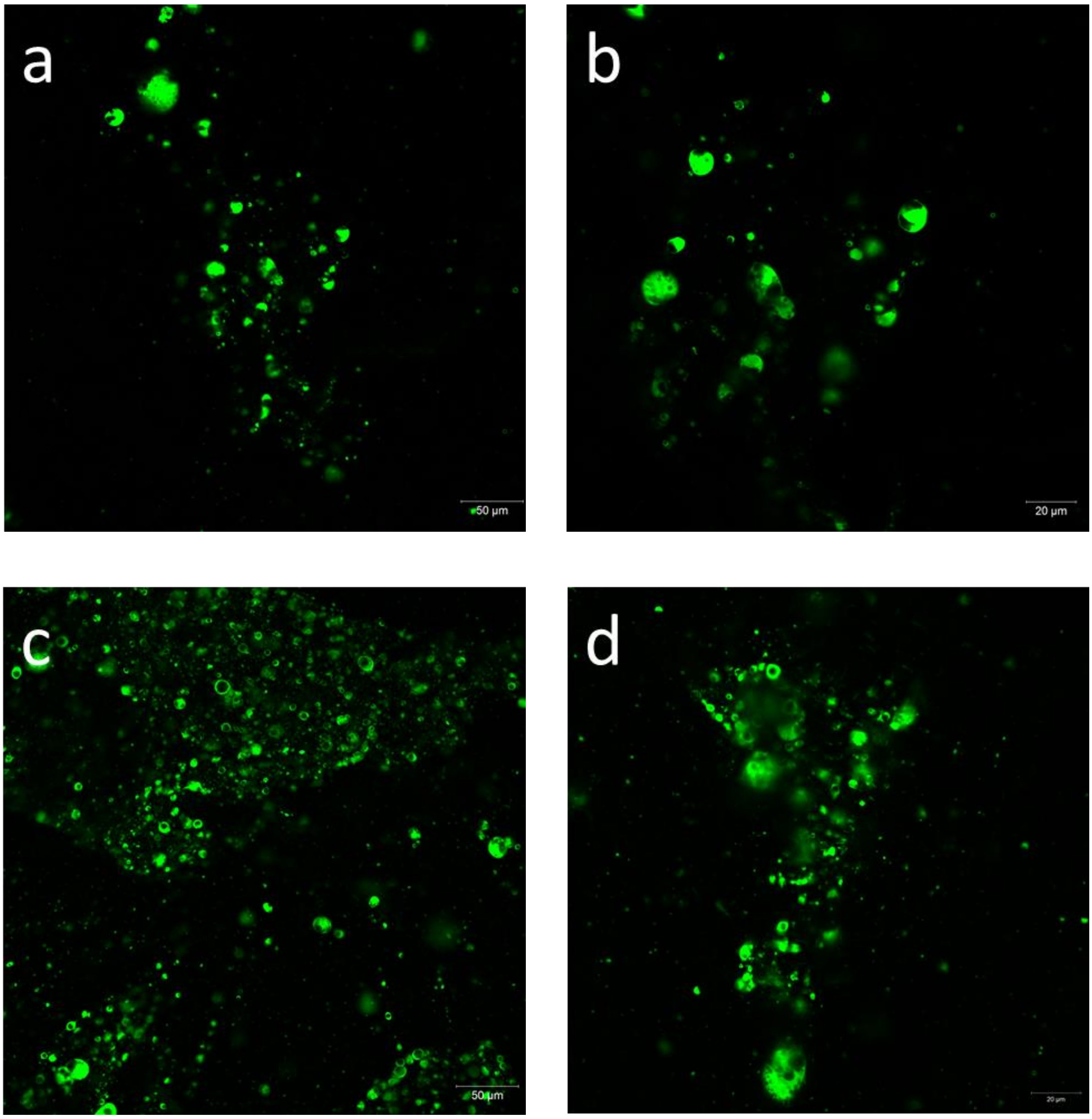

Figure S1. Selected 2D micrographs obtained by CSLM for CS-DES films, (a) F5/2 at 20x, (b) F5/2 at 40x, (c) F5/5 at 20x, and (d) F5/10 at 40x magnifications. The açaí oil encapsulated in the PECs appears in green, whereas the CS-DES based matrices appear in black.

Table S1. E' (MPa) at 25 °C and tan δ (°C) for the CS-based films.

Sample	E' (MPa) at 25 °C	tan δ (°C)		
		Peak$_1$	Peak$_2$	Peak$_3$
F0/0	211.3	-8.1	165.3	263
F5/0	2.65	2.2	188	254
F5/0.5	1.13	-9.2	72	252
F5/2	0.91	-4.5	88.5	254
F5/10	0.98	-3.4	86.3	254

Table S2. TGA and DTG data for CS-based films.

**Sample**	**T_{onset} (°C)**	**T_{peak} (°C)**	**Residue (%) at 700°C**
F0/0	241.3	294.2	32.44
F5/0	169.9	281.4	16.40
F5/0.25	201.8	256.1	12.59
F5/0.5	173.9	253.5	11.49
F5/1	192.8	252.9	10.98
F5/2	171.3	254.8	13.22
F5/5	174.6	246.3	12.38
F5/10	182.2	251.8	12.64
